# Epitranscriptomic mechanisms of androgen signalling and prostate cancer

**DOI:** 10.1016/j.neo.2024.101032

**Published:** 2024-07-20

**Authors:** Rodhan Patke, Anna E. Harris, Corinne L. Woodcock, Rachel Thompson, Rute Santos, Amber Kumari, Cinzia Allegrucci, Nathan Archer, Lorraine J. Gudas, Brian D. Robinson, Jenny L. Persson, Rupert Fray, Jennie Jeyapalan, Catrin S. Rutland, Emad Rakha, Srinivasan Madhusudan, Richard D. Emes, Musalwa Muyangwa-Semenova, Mansour Alsaleem, Simone de Brot, William Green, Hari Ratan, Nigel P. Mongan, Jennifer Lothion-Roy

**Affiliations:** a Biodiscovery Institute, University of Nottingham, UK; b School of Veterinary Medicine and Science, University of Nottingham, UK; c Department of Pharmacology, Weill Cornell Medicine, New York, NY, USA; d Department of Molecular Biology, Umea University, Umea, Sweden; e School of Biosciences, University of Nottingham, UK; f School of Medicine, University of Nottingham, UK; g Nottingham University NHS Trust, Nottingham, UK; h Research and Innovation, Nottingham Trent University, UK; i University of Zambia, Lukasa, Zambia; j Unit of Scientific Research, Applied College, Qassim University, Qassim, Saudi Arabia; k Institute of Animal Pathology, University of Bern, Switzerland; l Nottingham University Hospitals NHS Trust, Nottingham, UK

## Abstract

Prostate cancer (PCa) is the second most common cancer diagnosed in men. While radical prostatectomy and radiotherapy are often successful in treating localised disease, post-treatment recurrence is common. As the androgen receptor (AR) and androgen hormones play an essential role in prostate carcinogenesis and progression, androgen deprivation therapy (ADT) is often used to deprive PCa cells of the pro-proliferative effect of androgens. ADTs act by either blocking androgen biosynthesis (e.g. abiraterone) or blocking AR function (e.g. bicalutamide, enzalutamide, apalutamide, darolutamide). ADT is often effective in initially suppressing PCa growth and progression, yet emergence of castrate-resistant PCa and progression to neuroendocrine-like PCa following ADT are major clinical challenges. For this reason, there is an urgent need to identify novel approaches to modulate androgen signalling to impede PCa progression whilst also preventing or delaying therapy resistance.

The mechanistic convergence of androgen and epitranscriptomic signalling offers a potential novel approach to treat PCa. The epitranscriptome involves covalent modifications of mRNA, notably, in the context of this review, the N(6)-methyladenosine (m^6^A) modification. m^6^A is involved in the regulation of mRNA splicing, stability, and translation, and has recently been shown to play a role in PCa and androgen signalling. The m^6^A modification is dynamically regulated by the METTL3-containing methyltransferase complex, and the FTO and ALKBH5 RNA demethylases.

Given the need for novel approaches to treat PCa, there is significant interest in new therapies that target m^6^A that modulate AR expression and androgen signalling. This review critically summarises the potential benefit of such epitranscriptomic therapies for PCa patients.

## Mechanisms of prostate carcinogenesis and progression

Prostate cancer (PCa) is the second most common cancer affecting men and is most commonly diagnosed in patients over 65 years of age. PCa that develops before the age of 55 often has a more aggressive phenotype associated with poorer patient outcomes [1]. A number of risk factors for PCa have been identified, including age, genetic ancestry, and family history [2], as well as environmental and lifestyle factors such as diet and smoking [3,4]. There are significant differences in PCa incidence rates between genetic ancestries, with incidence twice as high in men of African ancestry compared to their age-matched white counterparts [5]. Luminal cells of the acini are considered the most common cell of origin for PCa, with acinar adenocarcinoma expressing androgen receptor (AR) accounting for ∼95 % of PCa cases [6]. Routine screening using the prostate specific antigen (PSA) test remains controversial and may contribute to overdiagnosis and thus overtreatment of PCa, both by detecting false positives and clinically insignificant PCa [7]. PCa most commonly metastasises to the bones of the pelvis and lumbar spine, as well as the regional lymph nodes, abdominal viscera and lungs [8], with a subsequent median survival time of approximately three years [9].

PCa spontaneously arising from prostate neuroendocrine cells is rare (observed in ∼2 % of patients), however in ∼25 % of patients with metastatic castrate-resistant prostate adenocarcinoma, neuroendocrine-like PCa (NEPC) can emerge following androgen deprivation therapy (ADT) [10]. NEPC cells lack AR expression and are associated with the loss of key tumour suppressor proteins including p53 and PTEN [11,12]. NEPC represents a key clinical challenge due to its highly aggressive phenotype and the absence of effective therapies [13].

### Current prostate cancer treatments

In recent years there has been increasing focus on personalised precision medicine to identify the underlying genetic mechanisms of cancer progression in individual patients, and to identify markers that will better predict response to therapy and facilitate selection of the most appropriate treatment plans to optimise outcomes [14]. Several treatment options are available for PCa patients, although many are associated with marked side effects [15]. Curative treatments include radical prostatectomy and radiotherapy for organ-confined disease. There is no curative treatment for metastatic PCa, although disease progression can be delayed by ADT. A major issue is the lack of a reliable method to distinguish patients with indolent PCa from those with high-risk aggressive tumours. Thus, the identification of patients who will develop clinically significant disease that will progress to lethal PCa remains a key clinical challenge.

**Fig. 1:**
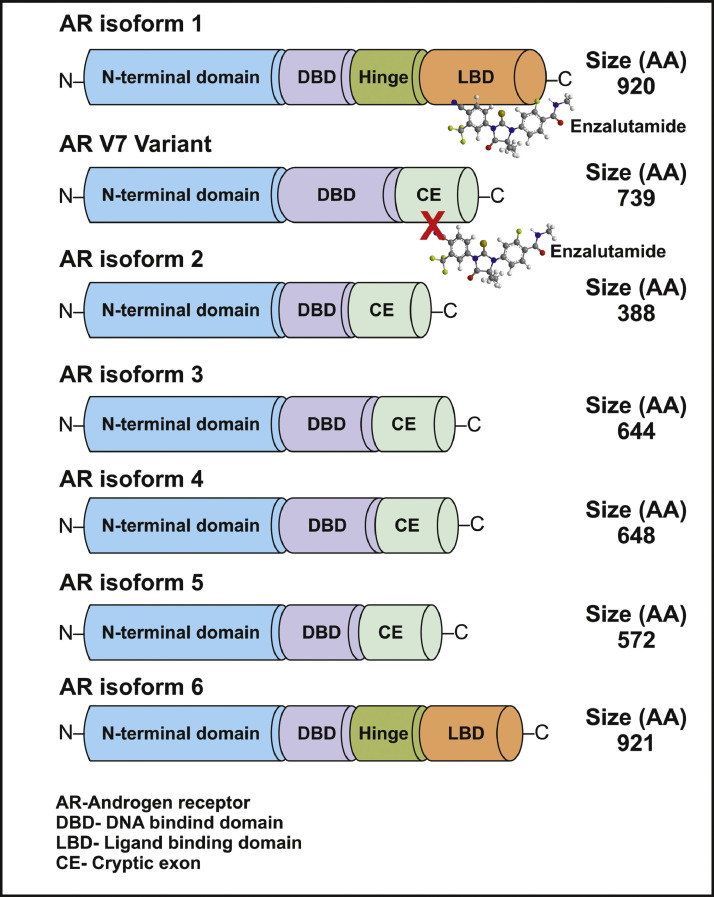
Structure of the androgen receptor and its splice variants. Schematic representation illustrating the comprehensive structure of the androgen receptor (AR), including annotated domains, amino acid lengths and splice variants.

**Fig. 2:**
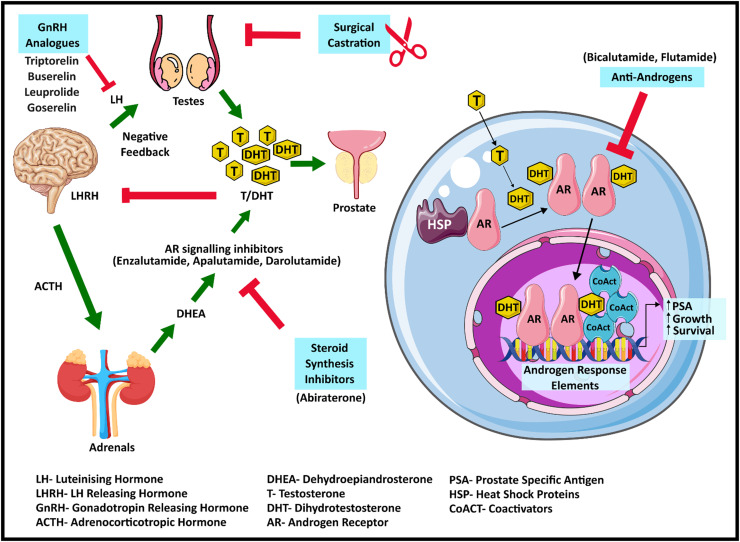
Therapeutic targets in androgen deprivation therapy (ADT) in prostate cancer. Demonstration of therapeutic targets targeted by ADT for treating prostate cancer, outlining the clinical molecular targets together with their respective analogues and antagonists.

Androgen hormones, primarily testosterone and dihydrotestosterone (DHT), are responsible for PCa carcinogenesis, stimulating cell proliferation and therefore the opportunity to acquire genetic mutations during cell division [16,17]. The AR (Fig. 1) plays a key role in altering PCa cellular proliferation and survival, thereby contributing to carcinogenesis and disease progression in PCa [17,18]. In advanced PCa, ADTs, which seek to deprive PCa cells of the androgen signalling that stimulates their proliferation and invasion [19], are standard of care (Fig. 2).

The AR is a ligand-dependent transcription factor encoded by the *AR* gene, and is a member of the steroid receptor family of nuclear receptors activated by testosterone and its more potent derivative DHT [20]. The AR is essential for normal embryonic and post-pubertal development and plays an essential role in the development and progression of PCa [21]. The AR recruits multiple epigenetic coregulators to initiate AR-regulated gene transcription [22] (Fig. 1).

ADTs block androgen signalling through distinct endocrine mechanisms, which all converge at the AR. The gonadotrophin hormone-releasing hormone (GnRH) agonists (e.g. triptorelin, buserelin, leuprolide, and goserelin) and antagonists (e.g. degarelix), together with steroid biosynthesis inhibitors (e.g. abiraterone acetate), reduce circulating androgen levels, thereby reducing AR activation. AR signalling inhibitors (ARSIs) such as bicalutamide, enzalutamide, apalutamide and darolutamide are AR antagonists that bind to the AR ligand binding domain and prevent AR-mediated transcriptional activation [23] (Fig. 2).

ADT is often effective initially, with tumour regression observed in ∼80 % of patients. However, in many cases ADT resistance emerges and progression to castration-resistant prostate cancer (CRPC) often occurs within approximately two years of treatment [24]. The AR and androgen signalling are now known to continue to play an important, but distinct, role in CRPC [25]. For this reason, ARSIs such as enzalutamide can benefit CRPC patients that have relapsed following chemotherapy [26], and progression-free survival in chemotherapy-naïve patients [27]. More recently these drugs have also been approved for use in non-metastatic CRPC as they improve metastasis-free survival [28].

PCa cells exploit a number of mechanisms to adapt to ADT and low circulating androgen levels, which are believed to contribute to the emergence of CRPC [29]. These include increased AR protein expression [30], expression of AR variants and *AR* gain-of-function gene mutations [[31], [32], [33]]. *AR* mutations can enable promiscuous activation of the AR by other steroids [[33], [34], [35]], or by enabling ARSIs such as enzalutamide and bicalutamide to function as agonists that activate AR-regulated transcription [36]. The increased expression of AR splice variants such as AR-V7 (Fig. 1) that lack the ligand binding domain targeted by ARSIs is also believed to contribute to CRPC [33]. Altered extragonadal androgen biosynthesis [37] and altered expression of transcriptional co-regulators are also implicated in CRPC [38].

Advanced disease is treated with systemic cytotoxic chemotherapy [19], using drugs such as docetaxel in combination with the corticosteroid prednisone, or radiotherapy. These have been demonstrated to improve survival rates when used alongside ADT [19,39]. More recent innovations in PCa treatment include the autologous immunotherapy Sipuleucel-T (approved in the USA), which uses a patient's own antigen-presenting cells activated by prostatic acid phosphatase (PAP) which, in turn, stimulates a T cell immune response to PCa cells [40]. To date, immunotherapy has shown limited patient benefit. The recent approval of the anti-PD-1 monoclonal antibody pembrolizumab [41], and theragnostic antibodies targeting the prostate specific membrane antigen (anti-PSMA) to deliver radioligand therapy, such as ^177^Lu-PSMA, have extended the therapeutic repertoire for advanced PCa [42]. Both pembrolizumab and ^177^Lu-PSMA-617 in combination are in phase Ib trial studies for CRPC patients (clinicaltrials.gov accession#: NCT03805594 date accessed: January 9, 2024). Furthermore, phase 3 VISION trials of ^177^Lu-PSMA-617 in patients with CRPC are ongoing, with safety analysis supporting up to six cycles of ^177^Lu-PSMA-617 treatment [43]. Despite the development of novel treatments for advanced PCa, there remains an urgent need to explore new mechanistic approaches to suppress androgen signalling and its associated disease progression.

### The epitranscriptome, androgen signalling, and prostate cancer

**Fig. 3:**
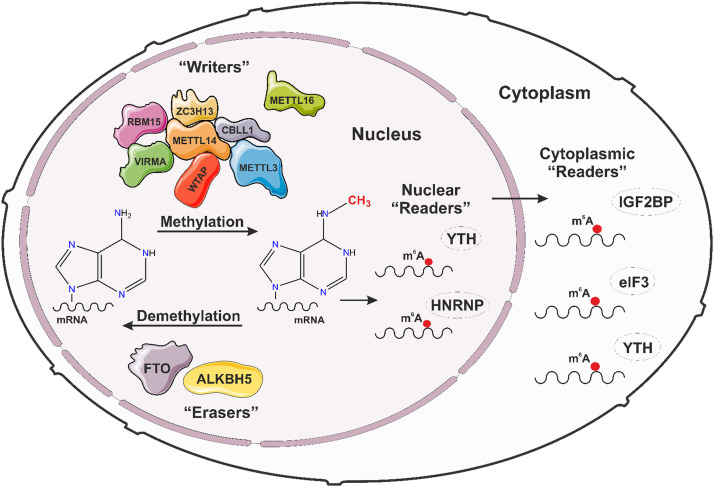
The components of the m^6^A methyltransferase complex, including m^6^A methylases, demethylases and “reader” proteins involved in dynamic mRNA modification. Representation of the components of the m^6^A methyltransferase complex comprising the methylases (“writer” proteins) and the demethylases (“eraser” proteins), together with the effector "reader” proteins that bind to m^6^A modifications both within the nucleus and cytoplasm of cells.

Given the importance of androgen signalling and AR expression in PCa progression, new therapies targeting the AR transcriptional machinery may represent feasible therapeutic targets. AR-mediated transcriptional activation depends on an extensive network of epigenetic coregulators [44]. Whilst the therapeutic potential of targeting AR epigenetic coregulators is well-recognised with associated clinical trials underway [45], more recently attention has been focussed on new therapies targeting RNA modifications, commonly referred to as the epitranscriptome. There are recently revealed mechanistic links between histone and RNA modifications [46] indicating crosstalk between epigenetic and epitranscriptomic signalling. The most common internal covalent modification of mRNA in eukaryotes is methylation of the N^6^ position of adenosine on cellular mRNA, also known as N^6^-methyladenosine (m^6^A) [47,48] (Fig. 3). The m^6^A mRNA modification is functionally conserved across species. We and others [[49], [50], [51], [52], [53], [54]] have identified roles for m^6^A in the regulation of gene expression, splicing, polyadenylation, RNA transportation, decay and translation [[55], [56], [57]]. The dysregulation of m^6^A has been shown to contribute to a number of diseases [[58], [59], [60], [61], [62]], including PCa development and progression [[63], [64], [65], [66], [67], [68]], with evidence supporting m^6^A as a potential target for novel therapies against PCa.

The m^6^A modification is dynamically regulated by the methyltransferase-like 3 (METTL3)-containing methyltransferase (“writer”) complex [[68], [69]] and the FTO and ALKBH5 demethylases (“erasers”) [70]. The presence of m^6^A is detected and interpreted by m^6^A binding proteins (“readers”) (Fig. 3). METTL3 expression has been found to be upregulated in PCa patients and PCa cell lines compared with non-malignant prostate cells, with METTL3 implicated in PCa progression, poorer prognosis and treatment resistance [[64], [68], [71], [72], [73]]. Interestingly, although many studies have identified an association between METTL3 and PCa development, as reviewed recently [74], little is known about the functional interaction between m^6^A, androgen signalling, and its impact on PCa carcinogenesis. We recently examined the clinical and mechanistic significance of METTL3, METTL14, WTAP, and CBLL1 in PCa patient specimens and cell lines [68,71]. We identified that METTL3 and CBLL1 were associated with poorer patient outcomes and reported higher mRNA and protein expression in PCa cell lines compared with non-malignant prostate cells [[68], [71]]. Most notably, highest expression was identified in the castrate-sensitive, androgen-responsive cell lines [71].

Interestingly, functional depletion of *METTL3* resulted in upregulation of AR, together with 134 AR-regulated genes [71], consistent with the findings observed following shRNA silencing of *METTL3* in PCa cell lines [64]. Our group have found that siRNA-mediated *METTL3* knockdown or METTL3-pharmacological inhibition significantly altered the basal and androgen-regulated transcriptome and splicing in PCa cell lines, including differential splicing of AR [68]. Notably, this resulted in a reduction of expression of AR variants lacking the ligand binding domain which are implicated in ARSI-resistance [[68], [71]]. In addition, mRNA expression of the *METTL3, METTL14,* and *WTAP* methyltransferase-complex components were positively correlated with AR mRNA expression [75]. These findings suggest a functional interaction between the methyltransferase complex component expression, m^6^A, and AR signalling in PCa, therefore supporting m^6^A as a potential therapeutic target for hormone-dependent PCa [71] .

AR translation was recently shown to be regulated by RNA binding proteins [76]. The m^6^A “reader” protein YTHDF3 and RNA binding protein G3BP1 coordinate the regulation of *AR* mRNA translation [76]. Under acute AR pathway inhibition stress by enzalutamide (Fig. 1), m^6^A-modified *AR* mRNA is recruited from actively translating polysomes to RNA-protein stress granules, resulting in reduced *AR* translation [76]. Thus ADT and AR signalling inhibition alter AR expression using an m^6^A-mediated mechanism, though whether this contributes to treatment-emergent NEPC remains unknown.

The emergence of resistance to ADT and ARSI treatments is a major clinical challenge, and therefore identifying mechanisms contributing to resistance that could be therapeutically exploited in PCa is urgently needed. Recent evidence supports a role for METTL3 in advanced metastatic disease. It has been observed that low METTL3 expression is associated with clinical factors of CRPC, which suggested that lower METTL3 function may influence resistance to ADT through AR-independent mechanisms [77]. A recent study identified increased m^6^A in CRPC compared with castrate-sensitive PCa patient tissue [73]. They also found METTL3 expression was increased after castration, activating the ERK pathway and contributing to a pro-oncogenic phenotype, including ADT resistance [73]. These studies highlight a role for m^6^A in ADT/ARSI therapy resistance and suggest METTL3 could be utilised both as a predictor of therapy response and as a therapeutic target. Further studies are now warranted to explore this mechanistically.

### The potential of targeting METTL3 therapeutically in prostate cancer

**Fig. 4:**
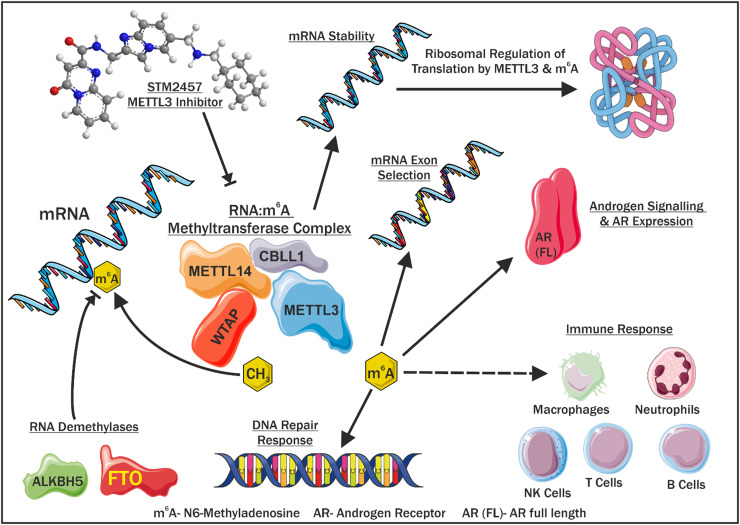
The m^6^A methyltransferase complex and its associated roles. Illustration of the dynamic interactions, catalytic activities, and functions of the m^6^A methyltransferase complex in mRNA selection, androgen signalling and AR expression, immune response, DNA repair response and translational regulation.

Given the identification of an association between altered METTL3, m^6^A, and cancer development, progression, and therapy resistance, there is considerable interest in METTL3 as a novel cancer therapeutic target. There are a number of small molecule METTL3 inhibitors currently being developed as recently reviewed [74]. A phase I clinical trial of STC-15, an oral small molecule METTL3 inhibitor, is currently underway for advanced malignancies that have failed standard of care treatments (clinicaltrials.gov accession #: NCT05584111, date accessed: 28 June 2024). STC-15, a derivate of STM2457, is a METTL3 inhibitor that has been reported to reduce acute myeloid leukaemia (AML) cell growth and increase survival in *in vivo* AML mouse models, with similar results identified in other cancer cell lines [78]. Preliminary phase I results on the therapeutic feasibility of METTL3 inhibition were recently reported [79], indicating m^6^A depletion and associated biological responses are achievable in patients. Our recent study identified that METTL3 inhibition reduced cellular proliferation in PCa cells [68] and thus the investigation of such METTL3-targeted therapies to benefit PCa patients is warranted (Fig. 4). While a deeper understanding of the complexity of the m^6^A-mediated mechanisms *in vivo* is required to identify any potential off-target effects, the adverse events reported to date were manageable [79]. The results of the ongoing clinical trials of the effect of METTL3 inhibition should provide valuable insights into the therapeutic potential of targeting METTL3 and m^6^A.

## Conclusions

Given the current major clinical challenges of the emergence of resistance to ADT and the progression to NEPC, there is an urgent need for novel therapeutic approaches. The increasing evidence that the METTL3 RNA methyltransferase represents a novel therapeutic target in PCa is promising. Supportive of this, it has been demonstrated that METTL3 expression is higher in PCa cells compared with non-malignant prostate cells, and that functional depletion of *METTL3* alters basal and androgen-regulated transcription [68,71] and inhibits PCa cell proliferation [64]. There is also increasing evidence of the importance of m^6^A in PCa and androgen signalling, suggesting that METTL3 inhibitors may be useful as an alternative approach to suppress androgen signalling, impair PCa cell proliferation, and potentially delay or prevent PCa progression and development of treatment resistance. Together these studies highlight the therapeutic potential of inhibition of m^6^A regulators, both as monotherapies, and in combination with current treatments for PCa.

## CRediT authorship contribution statement

**Rodhan Patke:** Writing – review & editing, Writing – original draft. **Anna E. Harris:** Writing – review & editing, Supervision. **Corinne L. Woodcock:** Writing – review & editing, Supervision. **Rachel Thompson:** Writing – original draft. **Rute Santos:** Writing – original draft. **Amber Kumari:** Writing – review & editing. **Cinzia Allegrucci:** Writing – review & editing. **Nathan Archer:** Writing – review & editing. **Lorraine J. Gudas:** Writing – review & editing. **Brian D. Robinson:** Writing – review & editing. **Jenny L. Persson:** Writing – review & editing. **Rupert Fray:** Writing – review & editing. **Jennie Jeyapalan:** Writing – review & editing. **Catrin S. Rutland:** Writing – review & editing. **Emad Rakha:** Writing – review & editing. **Srinivasan Madhusudan:** Writing – review & editing. **Richard D. Emes:** Writing – review & editing. **Musalwa Muyangwa-Semenova:** Writing – review & editing. **Mansour Alsaleem:** Writing – review & editing. **Simone de Brot:** Writing – review & editing. **William Green:** Writing – review & editing. **Hari Ratan:** Writing – review & editing. **Nigel P. Mongan:** Writing – original draft, Supervision, Funding acquisition. **Jennifer Lothion-Roy:** Writing – review & editing, Writing – original draft.

## Declaration of competing interest

The authors declare that they have no competing financial interests or personal relationships that could influence the work reported in this paper.
